# The regulatory roles of neutrophils in adaptive immunity

**DOI:** 10.1186/s12964-019-0471-y

**Published:** 2019-11-14

**Authors:** Yang Li, Wei Wang, Fan Yang, Yanan Xu, Chang Feng, Yong Zhao

**Affiliations:** 1 State Key Laboratory of Membrane Biology, Institute of Zoology, Chinese Academy of Sciences, University of Chinese Academy of Sciences, Beichen West Road 1-5, Chaoyang District, Beijing, 100101 China; 2 Department of Urology, Beijing Chaoyang Hospital, Capital Medical University, Beijing, China; 3 Institute for Stem Cell and Regeneration, Chinese Academy of Sciences, Beijing, China

## Abstract

**Abstract:**

Neutrophils have long been considered as cells playing a crucial role in the immune defence against invading pathogens. Accumulating evidence strongly supported the direct and indirect regulatory effects of neutrophils on adaptive immunity. Exogenous cytokines or cytokines produced in an autocrine manner as well as a cell-to-cell contact between neutrophils and T cells could induce the expression of MHC-II and costimulatory molecules on neutrophils, supporting that neutrophils may function as antigen-presenting cells (APCs) in respects of presenting antigens and activating T cells. In addition to the inflammatory roles, neutrophils also have the propensity and ability to suppress the immune response through different mechanisms. In this review, we will mainly highlight the heterogeneity and functional plasticity of neutrophils and the antigen-presenting capacity of different neutrophil subsets. We also discuss mechanisms relevant to the regulatory effects of neutrophils on adaptive immunity. Understanding how neutrophils modulate adaptive immunity may provide novel strategies and new therapeutic approaches for diseases associated with neutrophils.

**Graphical abstract:**

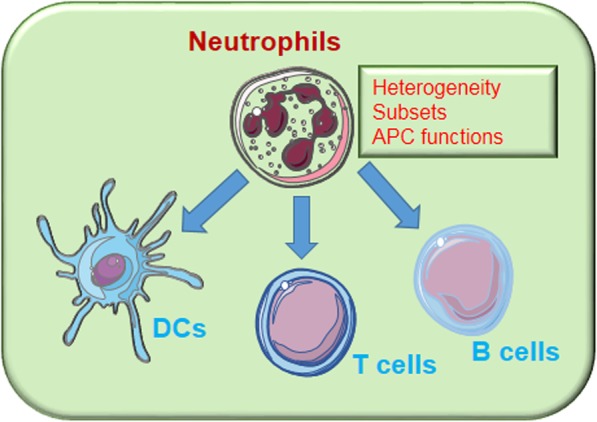

## Background

Neutrophils are one of the earliest identified members of innate immune cells and one of the main cell types involved in the inflammatory response, which are among the first defense line against invading pathogens. They play an important role in the immune defensive response against invading bacterial and fungal pathogens mainly by reactive oxidative species (ROS) generation, granule release and neutrophil extracellular traps (NETs) formation. However, a great deal of evidence shows that neutrophils also participate in the initiation and regulation of adaptive immunity [1–5]. Adaptive immunity is considerably important for individuals to control pathogen infection and tumor growth with specificity and immunological memory. It is noticeable, however, that innate immune cells provide signals for proliferation and activation of T and B cells to initiate adaptive immunity against self-antigens which would cause autoimmune diseases. Importantly, recent findings strongly indicate that neutrophils also act as APCs via direct interaction with T and B cells [2, 6–9]. The regulatory roles of neutrophils on adaptive immunity are somehow neglected for long. In this review, we summarized recent advances in neutrophils, which mainly focused on their plasticity in different microenvironments, as well as their role in regulating T and B cell activation and differentiation. In addition, the mechanisms employed by neutrophils to impact adaptive immune response are also discussed. We hope to promote our great attentions to the modulatory effects of neutrophils in adaptive immunity, which may be of significance for us to understanding the involvement of neutrophils in immune-related diseases.

### Subsets of neutrophils

Neutrophils are among the first defense line against invading pathogens, and play an important role in both innate and adaptive immunities. Accumulating data showed that neutrophils can switch phenotypes and display distinctive subpopulations (Table 1). Tsuda et al. first put forward the idea of the classification of neutrophils in mice. They showed that, in addition to the CD49d^−^CD11b^−^ resting neutrophils, there were existing at least two distinct subsets of neutrophils in mice [31]. The defined type 1 neutrophils (N1) and type 2 neutrophils (N2) are different in respects of cytokine and chemokine productions, promoting macrophage activation and the expressions of Toll-like receptors and surface antigens [31]. The CD49d^+^CD11b^−^ N1 neutrophils isolated from SCIDbg mice with mild systemic inflammatory response syndrome (SIRS) secrete the cytokine IL-12 and chemokine CCL3, while CD49d^−^CD11b^+^ N2 neutrophils isolated from SCIDbg mice with severe SIRS mainly produce IL-10 and CCL2. The CD49d^−^CD11b^−^ neutrophils from the uninfected SCIDbg mice failed to show cytokine and chemokine production [31]. Different neutrophil phenotypes are also confirmed in tumor-bearing mouse models. It is possible that various differentiation programs of neutrophils occur in distinct disease states depending on the cytokine milieu. Similar to tumor-associated macrophages (TAMs), tumor-associated neutrophils (TANs) also have different polarization states. Blockade of TGF-β skews mouse neutrophils differentiation to an anti-tumorigenic phenotype (N1), with more cytokine and chemokine production, lower levels of arginase and a stronger ability to kill tumor cells in vitro [32]. However, in the context of the tumor, TGF-β favours the accumulation of mouse pro-tumorigenic N2 neutrophils to promote the tumor growth [32]. During helminth infection, an alternatively activated mouse neutrophil (N2) population developed with a characteristic global transcriptional profile, which was distinct from LPS-stimulated mouse neutrophils (N1). Furthermore, mouse N2 neutrophils regulate macrophage differentiation with up-regulation of both M2 markers and adhesion molecules to mediate parasite damage and clearance during the secondary infection, which was dependent on IL-13 produced by neutrophils in mice [33]. Besides the role in the innate phase of the immune response, neutrophils also influence adaptive immunity by interacting with B cells. Neutrophils colonized peri-marginal zone (MZ) areas of the spleen through a noninflammatory process that became more prominent after birth and involved mucosal colonization by bacteria. In contrast to circulating neutrophils (conventional neutrophils, called N_C_ cells), mouse splenic neutrophils (B cell–helper neutrophils, termed as to N_BH_ cells), including N_BH_1 and N_BH_2 subsets, expressing B cell stimulating factors B cell-activating factor (BAFF), a proliferation inducing ligand (APRIL), IL-21 and B cell attracting chemokines CXCL12 and CXCL13. Thus N_BH_ neutrophils induced immunoglobulin class switching, somatic hypermutation and activating MZ B cells through both contact-dependent and contact-independent manners in mice. Compared with mouse N_C_ neutrophils, mouse N_BH_1 and N_BH_2 expressed higher CD11b and CD24 associated with inhibiting TLR signaling, and higher CD27, CD40L, CD86, CD95, MHC-I and MHC-II indicating immune activation. Moreover, mouse N_BH_1 and N_BH_2 expressed lower CD54, CD62L, CD62P, and CD102, which were adhesion molecules involved with endothelial adhesion and extravasation. In addition, the phenotype of mouse N_C_ neutrophils are CD15^high^CD16^high^, while mouse N_BH_1 cells are CD15^inter^CD16^inter^ and N_BH_2 neutrophils are CD15^low^CD16^low^. Rhesus macaque N_BH_1 expressed higher CD27, CD40L, CD86, CD95 and HLA-II, but lower CD24 compared with N_BH_2, accounting for the persistence of N_BH_1 but not N_BH_2 in inflamed spleens with a hypoplastic MZ in mice [34]. During *S. pneumoniae* infection, resident immature Ly6G^intermediate^ neutrophils in mouse spleens undergo emergency proliferation and mobilization from the splenic niche to increase the effector mature Ly6G^high^ neutrophil pool after pneumococcal stimulation [35]. In addition, mouse IL-23-treated neutrophils selectively produce IL-17A, IL-17F and IL-22 via STAT3 pathway and display a distinct gene profile in contrast to resting and LPS-treated mouse neutrophils [24]. Importantly, IL-17^+^ neutrophils can be detected in in the DSS-induced colitis mouse model [24]. Meanwhile, adoptive transfer of syngeneic IL-23-treated mouse neutrophils significantly promotes the severity of the pathogenesis in the DSS-induced colitis mouse model [24]. The IL-23 specifically induced the expressions of IL-17A, IL-17F and IL-22 in mouse neutrophils through the activation of mTOR and RORγT. Using a microbiota Ag-specific T cells–mediated colitis mouse model, they indicated a protective role of neutrophils and IL-22 in chronic colitis [36]. On the other hand, IL-33 stimulates neutrophils selectively producing IL-4, IL-5, IL-9 and IL-13 through c-Jun N-terminal kinase- and nuclear factor-κB-dependent pathways with a distinctive gene expression profile. Importantly, these newly-identified neutrophil subpopulation can be detected in an ovalbumin (OVA)-induced allergic asthma mouse model [25]. Adoptive transfer of syngeneic IL-33-treated mouse neutrophils significantly increased the severity of the lung pathogenesis in an OVA-induced allergic asthma mouse model [25]. These studies confirm the presence of neutrophil functional plasticity and polarization in vitro and in vivo. But the inducing factors, intracellular signals, the phenotype characteristics and the biological significance of the differentially polarized neutrophils need to be deeply explored in the future. Whether human neutrophils display similar functional polarizations as mouse neutrophils and whether distinctive neutrophils present in vivo do need to be studied in the future.

**Table 1:** Neutrophils treated with different stimulations display different phenotypes and regulatory function on adaptive immunity

Treatment/source/subsets	Characteristics	Regulation on adaptive immunity	Species	Refs
GM-CSF, IFN-γ, IL-3	MHC-II, CD80, CD86, CD83	Undetected	Human	[10]
GM-CSF, IFN-γ	MHC-II, CD80, CD86	Undetected	Human	[11]
GM-CSF, IFN-γ, IL-4, TNF-α and M-CSF	Macrophage-like neutrophils with HLA-DR	Undetected	Human	[12]
GM-CSF, IFN-γ or combination	MHC-II, CD80, CD86 and CD83	To present peptide antigen to CD4^+^ T cells	Human	[13–15]
GM-CSF + IL-4 + TNF-α	HLA-DR, HLR-DQ, CD80, CD86 and CD40	Stimulate T cell proliferation	Human	[16]
Patients with acute infection	CD83	Undetected	Human	[17]
Patients with chronic inflammatory diseases	MHC-II, CD80/86	Undetected	Human	[17]
Patients with Wegener’s granulomatosis	MHC-II, CD80/86	Undetected	Human	[18, 19]
Peritoneal cavity after i.p. administration of glycogen	MHC-II	To present the lysozyme antigen to T cells	Mouse	[20, 21]
Peritoneal cavity after i.p. injection of Fusobacterium nucleatum	Undetected	Stimulate T cell proliferation	Mouse	[22]
GM-CSF	MHC-II, CD11c, CD80, CD86	To present antigens to CD4^+^ T cells	Mouse	[23]
N(IL-23) (IL-23-stimulated)	Th17 cytokines	Undetected	Mice	[24]
N(IL-33) (IL-33-stimulated)	Th9 cytokines	Undetected	Mice	[25]
N(LPS) (LPS-stimulated)	TNF-α, IL-6	Undetected	Mice	[24]
GM-CSF, IFN-γ, and TNF-α in sepsis patients	CD40, CD64, CD86	Undetected	Human	[26]
Synovial fluid of patients with RA, co-cultured with autologous T cells	MHC-II, CD14, CD64, CD83	Undetected	Human	[27]
Peritoneal cavity after TG injection, co-cultured with purified CD4^+^ T cells	MHC-II, CD86	Present peptides to T cells; Stimulate T cell proliferation and cytokine production	Mice	[28, 29]
The inflamed colon in colitis mouse	MHC-II, CD86	Stimulate T cell proliferation and cytokine production	Mice	[30]
CD49d^+^CD11b^−^ N1 (from MRSA-resistant mice)	IL-12, CCL3, TLR2, TLR4, TLR5, TLR8	Th1 response	Mice	[31]
CD49d^−^CD11b^+^ N2 (from MRSA- sensitive mice)	IL-10, IL-4, CCL2, TLR2, TLR4, TLR7, TLR9	Th2 response	Mice	[31]
CD49d^−^CD11b^−^ neutrophils (from naïve mice)	TLR2, TLR4, TLR9	no chemokines and cytokines	Mice	[31]
N1 TAN (blockade of TGF-β in tumor microenvironment)	Immuno-activating cytokines and chemokines	anti-tumorigenic; increasing CD8^+^ T cell activation	Mice	[32]
N2 TAN (stimulated by TGF-β in tumor microenvironment)	Undetected	Pro-tumorigenic; decreasing CD8^+^ T cell activation	Mice	[32]
Parasite-primed (helminth infection)	IL-13, IL-33	macrophage activation	Mice	[33]
NCs in spleen (infected by bacteria)	IL-12	acting as APC-like cells	Mice	[31]
CD15^inter^CD16^inter^ N_BH_1 (microbes infection)	IL-21, BAFF, APRIL, CXCL12, CXCL13; N_BH_1 expresses higher CD27, CD40L, CD86, CD95 and MHC-II, than N_BH_2	B cell activation	Mice	[34]
CD15^low^CD16^low^ N_BH_2 in spleen (microbes infection)	IL-21, BAFF, APRIL, CXCL12, CXCL13; N_BH_2 expresses higher CD24 than N_BH_1	B cell activation	Mice	[34]

### Neutrophils acquire the APCs-like characteristics

Dendritic *cells* (DCs) are the major APCs that can initiate naive T cell response. Macrophages and B cells also express MHC-II and costimulatory molecules and are capable of activating effector or memory CD4^+^ Th cells. Vascular endothelial and some epithelial cells also express major histocompatibility complex II (MHC-II) and costimulatory molecules usually induced by IFN-γ as non-professional APCs, physiologic significance of their antigen presentation ability to CD4^+^ T cells is still needed to be clarified. In the early days, neutrophils were considered to be APCs, as evidence by the expression of MHC-II and costimulatory molecules on neutrophils or T cell response mediated by super-antigens of neutrophils without intracellular processing [37]. Freshly isolated human resting neutrophils express low levels of MHC-II and costimulatory molecules and are incapable of activating naive CD4^+^ T cells in a mixed lymphocyte reaction [38]. Neutrophils may acquire the feature of APCs under some specific circumstances.

Mouse neutrophils isolated from peritoneal cavity after i.p. administration of glycogen shows detectable levels of MHC-II on their surface [20]. The MHC-II is essential for effective presentation of the lysozyme antigens to antigen-primed T cells [21]. Neutrophils induced in the peritoneum of mice by i.p. injection of Fusobacterium nucleatum stimulates the proliferation of allogeneic T cells [22]. In addition, neutrophils obtained from peritoneal cavity after i.p. administration of thioglycollate present MHC-II-restricted peptides and induce T cell proliferation [28]. Mouse immature and mature neutrophils from bone marrow treated with GM-CSF rather than other growth factors also acquired a DCs-like phenotype. These cells up-regulated the expression of DC markers, such as CD11c, MHC-II, CD80, and CD86, but preserved neutrophil markers like Ly6G, CXCR2, and 7/4 (a neutrophil marker). Murine neutrophils also acquire ability to present foreign antigens to CD4^+^ T cells retaining ability of bacterial killing [23]. In the presence of GM-CSF, IFN-γ and IL-3, neutrophils could express MHC-II and costimulatory molecules [10]. The expressions of MHC-II, CD80 and CD86 on the surface of neutrophils could be induced by culturing healthy human neutrophils with GM-CSF and IFN-γ [11]. CD15^+^CD14^−^ cell population from peripheral blood mononuclear cells (PBMCs) cultured with GM-CSF + IFN-γ + IL-4 + TNF-α, subsequently with M-CSF alone induce macrophages-like neutrophils with HLA-DR expression [12]. Treatment with GM-CSF or IFN-γ increases the expression of MHC-II on human neutrophils, but no MHC-II has been detected in untreated samples [13, 14]. Neutrophils from healthy human donors treated in vitro with GM-CSF, IFN-γ or combination express MHC-II, CD80, CD86 and CD83, which always considered as DC markers. Meanwhile typical markers of neutrophils like CD66b, CD15, and integrin including CD11a/b/c are preserved. These cells also acquire the ability to present peptide antigen to CD4^+^ T cells via MHC-II [15]. The highly purified human lactoferrin^+^ neutrophils could be reprogramed to DCs-like cells by the combination of GM-CSF, IL-4 and TNF-α treatment. Although the human neutrophils-derived DCs stimulate T cell proliferation by up-regulating expression of HLA-DR, HLR-DQ, CD80, CD86 and CD40, the freshly isolated human neutrophils do not have this feature [16]. To assess the presence of these cells in vivo, neutrophils were isolated from patients with acute bacterial infection or chronic inflammatory diseases. In patients with acute infection, 80% of neutrophils expressed CD83 but not MHC-II and CD80/86 expression. In patients with chronic inflammatory diseases, neutrophils expressed MHC-II, CD80/86 rather than CD83. This suggests different signals regulating CD83 and MHC-II expressions [17]. Moreover, neutrophils of patients with Wegener’s granulomatosis also acquire DCs-like characteristics expressing MHC-II, CD80 and CD86 [18, 19]. The neutrophils in sepsis patients display a APCs-like phenotype with high expressions of CD40, CD64 and CD86 [26]. Thus, inflammatory environments likely promote the antigen-presenting ability of neutrophils.

T cells play a role in shaping neutrophils to differentiate into APCs [39]. MHC-II up-regulation on neutrophils depends on co-culture with T cells and antigens, but TLR ligands stimulation is not sufficient. After phagocytosis of gram-positive and gram-negative bacteria, neutrophils produce the corresponding ligands to activate unconventional T cells, which in turn promote the differentiation of neutrophils into APCs by GM-CSF, IFN-γ, and TNF-α secretion. Neutrophils from synovial fluid of inflamed joints of patients with rheumatoid arthritis express the enhanced levels of CD14 and CD64, and acquired MHC-II and CD83 when co-cultured with autologous T cells or T cell lines probably induced by T cells-derived cytokines [27]. Neutrophils isolated from peritoneal cavity of mice after thioglycollate (TG) injection do not express CD86 and MHC-II, but after co-culture with purified CD4^+^ T cells for 2 h, these molecule expressions on neutrophils are up-regulated. After incubating with OVA, these neutrophils are co-cultured with Carboxyfluorescein diacetate, succinimidyl ester (CFSE)-labelled CD4^+^ T cells for 4 days. CCFSE dilution shows that T cell proliferation can be stimulated by highly purified neutrophils. These CD4^+^ T cells produce cytokines IFN-γ and IL-17, indicating they are differentiated towards Th1 and Th17 [29]. In a mouse model of chronic colitis, colonic neutrophils with enhanced expression of MHC-II and CD86 acquired APC function that stimulated T cell proliferation and cytokine productions after co-cultured with CD4^+^ T cells [30]. Thus, in addition to the inflammatory microenvironments, T cells may be also closely involved in the process to gain the antigen-presenting ability of neutrophils.

### Neutrophils function as APCs

It was identified that neutrophils could carry antigens to lymph nodes (LNs) like professional APCs. A subset of human or mouse neutrophils expressing CCR7 was demonstrated. In vitro, the CCR7 rapidly expresses on stimulated neutrophils, which migrates in response to the CCR7 ligands CCL19 and CCL21. Injection of complete Freund adjuvant (CFA) recruited neutrophils to draining LNs (dLNs) in wild-type mice but not in CCR7-deficient mice [40]. Neutrophils can capture bacilli in peripheral tissues and transport them to the lymphoid organ mainly through afferent lymphatics in pathogen-induced inflammation [41]. Many of neutrophils are recruited in draining popliteal LNs after OVA is injected into the footpad of OVA/CFA immunized mice by means of lymphatic vessels, which provides new evidence about the roles of neutrophils in adaptive immunity in vivo [42]. However, following induction of sterile inflammation by photodynamic therapy (PDT), neutrophils migrates to dLNs mainly through crossing high endothelial venules (HEVs) similar to naive T cells. However, they depend on CXCR2 and IL-17A-induced CXCL2 expression in nonhematopoietic cells for entry, not CCR7 [43]. An early study showed that phagocytosed antigens by neutrophils can be cross-presented to CD8OVA1.3 T hybridoma cells via proteasome independent pathway [44]. Another study showed that neutrophils cross-primed OT1 CD8^+^ T cells by OVA treatment in vivo. This process was dependent on TAP and proteasome which was as efficient as in macrophages [45]. After intradermal injection of modified vaccinia Ankara virus, antigen-specific CD8^+^ T cells were localized in the bone marrow and dLNs of mice [45].

Freshly isolated human neutrophils can present antigens to memory CD4^+^ T cells through HLA-DR. In ex vivo assays, neutrophils from dLNs and spleen of immunized rhesus macaques can present vaccine antigen to autologous antigen-specific memory CD4^+^ T cells [38]. Previous in vitro assays always include immature neutrophil precursors. Under growth factor and cytokine stimulation, these precursors differentiate into functional distinct subset of neutrophils with APC capacity. But mature neutrophils were isolated with high purity, side-by-side comparison with DCs and monocytes was performed for their capacity of antigen presentation. Their antigen presenting ability is somehow lower than DCs and monocytes. Considering that they exist in large quantities in dLNs, spleens and inflammatory tissues during infections, their low-efficient capacity for antigen presentation to memory T cells may be overcome due to their large cell numbers. Therefore, the overall anti-presenting ability and regulatory function of neutrophils should not be neglected in certain situations.

### Mechanisms involved in the antigen presenting capacity of neutrophils

**Fig. 1:**
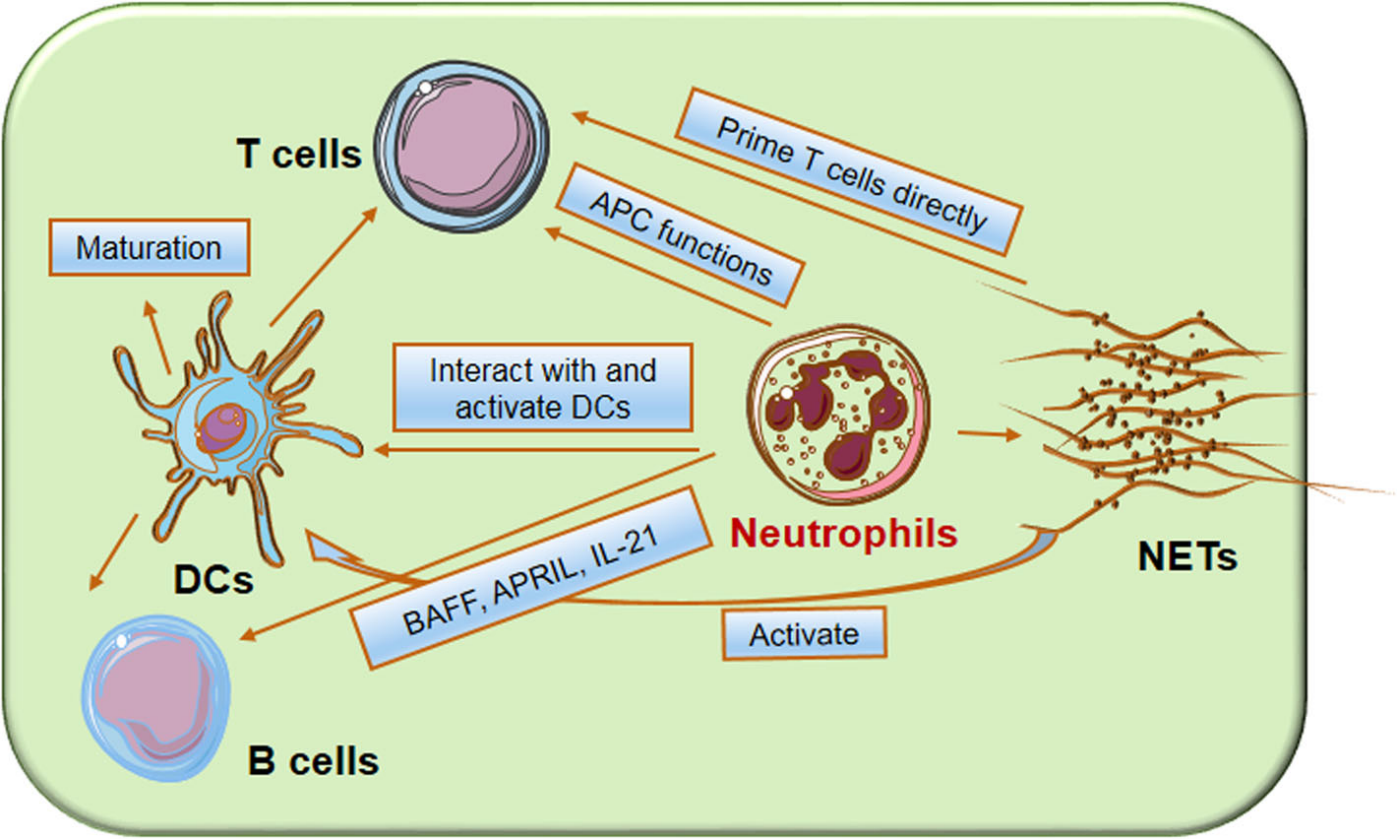
The regulatory effects of neutrophils on adaptive immune cells. Neutrophils regulate the adaptive immunity in direct and indirect manners. Neutrophils can migrate to dLNs and regulate T cell activation. Neutrophils promote T cell response via secreting the chemokines that attract T cells to the site of inflammation. Neutrophils can deliver antigens to DCs and make DCs more effective initiators of naive CD4^+^ T cell activation. Neutrophils in the dLNs localizing close to B cells accelerate plasma cell generation and antibody production associated with BAFF. Neutrophils in the spleen induce immunoglobulin class switching, somatic hypermutation and antibody production by activating MZ B cells through the productions of cytokines BAFF, APRIL and IL-21. NETs are composed of nuclear components such as DNA and histones and are decorated by proteins such as MPO and neutrophil elastase. NETs produced by neutrophils impact adaptive immunity by influencing DC maturation. NETs-stimulated pDCs via TLR9 induce anti-neutrophil cytoplasmic antibody production and related autoimmunity

Resting neutrophils express low levels of MHC-II and is incapable of activating naive CD4^+^ T cells in mixed leukocyte reaction (MLR) [38], neutrophils may gain the APC function possibly via the following mechanisms (Fig. 1). Freshly isolated normal human neutrophils contain CD80, CD86 [46] and MHC-II [47] mainly in cytoplasm, which could be translocated to the cell surface upon stimulation [48]. However, exogenous cytokines or cytokines produced in an autocrine manner induce MHC-II and costimulatory molecule expressions on neutrophils. As described earlier, cytokines such as GM-CSF, IFN-γ, IL-3, IL-4, and TNF-α could induce the expression of MHC-II and costimulatory molecules by neutrophils. Among these cytokines, IFN-γ seems to play the pivotal role [2, 39]. Recently, research has confirmed that IFN-γ induce MHC-II expression by the induction of the independent promoter IV, which regulates MHC-II transcription regulator class II transactivator [49]. In addition, IFN-γ in resting neutrophils has been detected [47], which can be released during culture or stimulation [50]. Therefore, IFN-γ may function in an autocrine manner to control MHC expression. On the other hand, neutrophils may gain the antigen-presenting ability in a cell-to-cell contact manner between neutrophils and T cells. As mentioned above, neutrophils isolated from mouse peritoneal cavity after TG injection did not express CD86 and MHC-II, but these molecule expressions are up-regulated after co-culture with purified CD4^+^ T cells for 2 h. However, this process is abolished when neutrophils and T cells are separated by a transwell system [29]. The receptors which mediate the interaction remains unclear. As we discussed earlier, memory T cells are efficient to stimulate neutrophils differentiation, which may be linked with the higher levels of receptors by memory T cells, such as intercellular adhesion molecule I (ICAM-1) [51]. The detailed mechanisms associated with the antigen processing and the type of antigens that neutrophils processing and presenting is still unclear.

### The regulatory effects of neutrophils on T cells

#### Direct effect of neutrophils on T cells

Neutrophils regulate T cell function through direct effects or affecting the function of DCs. Neutrophils migrate to dLNs and regulate T cell activation [43, 52–54]. Neutrophils have the ability to carry antigens to LNs in a CCR7-depend manner [40–42]. One study showed that neutrophils in inflamed skin egressed to dLNs via lymphatic vessels, which was dependent on CD11b and CXCR4 but not CCR7. Inhibiting their migration to dLNs reduced T cell proliferation in dLNs. The positive effect of neutrophils on T cells was related to up-regulated expressions of MHC-II, CD80 and CD86 by mouse neutrophils [55]. In addition, mouse neutrophils could carry antigens from the dermis to the bone marrow, initiating a source of memory CD8^+^ T cells, which is depend on CCR1 [56]. Meanwhile, depletion of bone marrow phagocytic cells abrogated the priming of CD8^+^ T cells in the bone marrow [56]. Another study identified that, upon LPS stimulation, neutrophils in the spleen migrated from the red pulp and MZ to the area of the white pulp where T cells reside in a CD14-dependent manner and interacted with naive T cells in mice [57]. Neutrophils also promote T cell response via secreting the chemokines that attract T cells to the site of inflammation, which has been demonstrated in murine models of contact hypersensitivity with production of CCL1, CCL2, and CCL5 [58] as well as allospecific CD8^+^ T cell responses with production of MCP-1 [59]. Highly purified human neutrophils could produce Th17 cells-recruiting chemokines CCL2, CCL20 and Th1 cells-recruiting chemokines CCL20, CCL19 after stimulation with LPS and IFN-γ [53]. It is revealed the relationships between neutrophils and T cells in infection models. In IL-10^−/−^ mice, gastric *Helicobacter* infection elicited a severe chronic gastritis and a greatly enhanced *Helicobacter*-specific Th1 immune response. But neutrophil depletion in these mice can decrease the Th1 immune response [60]. Neutrophil depletion by anti-Gr-1 antibody at 1 day before *L. pneumophila* infection significantly increases the susceptibility of mice to pneumonia and changed the bias of Th cell polarization from Th1 to Th2 [61]. Moreover, yeast treatment in vitro increases secretion of IL-12 and IL-10 by neutrophils. Neutrophil ablation early in the course of Th1-associated *Candida albicans* infection models also impairs Th cell polarization to Th1 and increases the susceptibility to infections [62].

#### Indirect effect of neutrophils on T cells via DCs

Neutrophils also regulate T cell immune response by affecting the function of DCs. Previous research indicated that neutrophils directly interacted with DCs to up-regulate TNF-α expression in DCs and activated DCs [63]. Co-culture with human neutrophils led to up-regulation of membrane CD86 and HLA-DR expressions on DCs via a cell contact-dependent mechanism, thus elicit antigen-specific T cell responses [64]. Moreover, in vitro co-culture of human neutrophils and DCs shows that they interact with each other by DC-SIGN on DCs binding to Mac-1 on neutrophils [65–67]. This kind of interaction can be found in vivo in colonic mucosa from patients with Crohn’s disease. The activated neutrophils also promote DC maturation via this interaction, which enhances the paracrine effect on DCs by neutrophils. The subsequent T cell response was also enhanced following this interaction [65]. *Mycobacterium tuberculosis* infection leads to transient accumulation of neutrophils in lungs at early stage of infection with subsequent recruitment of DCs to lungs in mice [68]. Depletion of neutrophils increases cell number of DCs in lungs, but results in their decreased migration to dLNs and delays activation and proliferation of naive Ag-specific CD4^+^ T cells in dLNs of mice [68]. In vitro assays also show that DCs acquired M. tuberculosis through uptake of infected neutrophils but not pathogens directly have better migratory ability, which results in robust CD4^+^ T cell activation in dLNs. These observations demonstrate the mechanisms of neutrophils in promoting adaptive immunity by delivering antigens to DCs and making DCs more effective initiators of naive CD4^+^ T cell activation [68].

### Effect of neutrophils on T cells via NETs and myeloperoxidase (MPO)

It is reported that neutrophils released neutrophil extracellular traps (NETs) to kill pathogens [69]. NETs-mediated T cell activation demonstrates a novel link between neutrophils and adaptive immune responses (Fig. 1). For instance, NETs released by human neutrophils could directly prime T cells by reducing their activation threshold with up-regulation of CD25 and CD69 on T cells, and NETs/cell contact and TCR signalling are needed. NETs-mediated priming increased T cell responses to antigens and even to suboptimal stimulus. Human peripheral blood mononuclear cells (PBMCs) co-culturing with NETs can also induce T cell activation and proliferation with IFN-γ and IL-17 productions [70]. Moreover, NETs impact adaptive immunity by influencing DC maturation. NETs alone has no discernable effect on monocyte-derived DCs (moDCs) after co-culture in vitro, but they down-regulated the maturation of moDCs stimulated by LPS with less HLA-DR, CD80, CD86 and CD83 expressions on DCs and down-regulated inflammatory cytokine productions. During DC maturation, NETs inhibit the capacity of DCs to induce T cell proliferation and modulate CD4^+^ T cell polarization by promoting the production of Th2 cytokines and reducing Th1 and Th17 cytokines. These findings reveal that neutrophils regulate DC maturation and thereby participate in the control of adaptive immune response [71]. MPO is an important granular protein for intracellular microbial killing by neutrophils. In vivo depletion or inhibition of MPO enhanced T cell responses in LNs with enhanced skin delayed-type hypersensitivity (DTH) and antigen-induced arthritis. In vitro assays showed that neutrophils-derived MPO inhibited DC activation by generation of reactive intermediates [72].

### The regulatory effects of neutrophils on humoral immune response

Neutrophils are confirmed as an effector cells in regulating B cell immune responses. The activating neutrophils, which are responsible for capturing and transporting circulating bacteria to the splenic MZ, promote B cells to initiate T cells-independent immune responses [73]. Human neutrophils stimulated by G-CSF express BAFF, which is important for B cell maturation and survival [74]. The recruited neutrophils in the dLNs localizing closely to B cells accelerated plasma cell and antibody generation associated with production of BAFF [75]. Reduction of neutrophil functions significantly diminishes plasma cell formation. Interestingly, neutropenic lysozyme 2-diphtheria toxin A mice exhibited striking emergency granulopoiesis and amplified neutrophil recruitment to the dLNs that is dependent on IL-17-induced prostaglandin activity [75]. Additionally, another cytokine APRIL stimulates B cell activation, while overexpression of APRIL could induce B-cell neoplasia. In B cell lymphoma, human neutrophils, constitutively producing APRIL and infiltrating the tumor tissue, are the main cellular source of APRIL [76]. Under homeostatic conditions, neutrophils are detectable in the perifollicular area of spleens and mLNs but not other lymphoid organs. IL-10 produced by splenic sinusoidal endothelial cells which closely locate to perifollicular neutrophils induce their B cell-helper function. These cells but not circulating neutrophils induce immunoglobulin class switching, somatic hypermutation and antibody production by activating MZ B cells after co-culture through the production of cytokines BAFF, APRIL and IL-21. Blocking these molecules by fusion protein or antibody abolishes IgM production and impairs the induction of IgG2 and IgA by MZ B cells [34].

In addition, NET structures are highly immunogenic such to trigger adaptive immune response relevant to autoimmunity. Uploaded with NET components, DCs could be activated, which subsequently induces anti-neutrophil cytoplasmic antibody production and related autoimmunity. DNase I treatment disrupted the activation of DCs, suggesting that intact NETs are needed for antigenicity of cytoplasmic proteins [77]. Neutrophils also participate in the pathogenesis of autoimmune disease, such as systemic lupus erythematosus (SLE), through NETs-mediated B cell activation. In SLE patients, self-DNA and antimicrobial derived from NETs could stimulate plasmacytoid DCs (pDCs) via TLR9 and served as autoantigens to trigger B cell activation. Neutrophils from SLE patients release more NETs than those from healthy donors, which is further stimulated by autoantibodies [78]. Another research showed that SLE pathogenesis had been linked to the increased production and/or bioavailability of IFN-α and associated alterations in DC homeostasis. NETs released by neutrophils containing DNA, LL37 and HMGB1 could activate pDCs to produce high levels of IFN-α in a DNA- and TLR9-dependent manner [79]. The above data establish a link between neutrophils, pDC activation and autoimmunity in SLE and indicate an important role of neutrophils in the disease pathogenesis via NETs. These findings provide new potential targets for the treatment of SLE.

### The negative regulatory effects of neutrophils in immunity

Although the evidence that the inflammatory roles of neutrophils have been well recognized for long, neutrophils indeed play a direct suppressive role on proinflammatory cytokine productions through different mechanisms. Neutropenia induced by chemotherapy or others will lead to the loss of the inhibitory propensity of neutrophils and potentially resulting in cytokinemia [80]. Studies with neutrophil deletion in PMN^dtr^ mice by injection of diphtheria toxin suggest that neutrophils were essential to protect the host from LPS-induced lethal inflammation in an MPO-dependent manner [81]. The neutrophils have an important negative role in the CD4^+^ T cells and B cell responses to three protein antigens, including hen egg white lysozyme, OVA, and listeriolysin O. Neutrophils migrate into the dLNs after immunization with proteins in any one of three adjuvants. After neutrophil depletion by antibodies for only 24 h or in genetically neutropenic mice, CD4^+^ T cell polarization to Th1 or Th2 is significantly enhanced by antigen immunization with CFA, IFA or alum. Neutrophils established brief contact with DCs and macrophages, and subsequently weakened the quality of DC-T cell interaction. Nevertheless, the exact mechanisms need to be clarified [82]. Acute graft-versus-host disease (aGVHD) protection relies on incoming IL-10^+^ neutrophils from G-CSF-treated donor spleen cells in mice [83]. These neutrophils had high phagocytic capacity and peroxide production, low MPO activity, cytoplasmic granule content as well as low expression of MHC-II, costimulatory molecules, arginase-1, IFN-γ, IL-17F, IL-2 and IL-12, which account for the regulation of regulatory T cell generation [83].

The immunosuppressive function of neutrophils has also been noted in several infection models. Neutrophils instructed by infected reservoir DCs produce IL-10 during mycobacterial infection and specifically shut-down Th17 cells through their IL-10 receptor [84]. At later time of intracellular Brucella infection, *Brucella abortus* is killed more efficiently in the absence of PMNs than in their presence, which is concomitant to promote the higher recruitment of monocytes and DCs, as well as significant activation of B and T cells and a balance of Th1 over Th2 response [85]. In an Aspergillus infection model, neutrophils show the propensity to suppress proinflammatory cytokine production through different mechanisms. For example, the modulation of IL-1β production by Aspergillus is cellular contact-dependent with the involvement of complement receptor 3. Inhibition of TNF-α is cell contact-independent and mediated by secreted MPO through the TLR4 pathway [86]. Furthermore, there are two waves of neutrophils entering dLNs after immunization. Both two waves of neutrophils depended on prostanoids to enter dLNs, as indomethacin treatment blocked both waves of neutrophil entry. Neutrophil depletion results in increased CD4^+^ T cell response by IL-2 or IFN-γ production and the increased T cell response in distant dLNs. In G-CSFR^−/−^ mice with few circulating neutrophils, similar results are found as enhanced spreading of T cell responses to distant dLNs. COX1 and COX2 deficiency or prostanoids blocked by indomethacin results in enhanced T cell responses and spreading to distant dLNs, suggesting the mechanism for neutrophils-mediated T cell regulation is dependent on prostanoids in mice [54]. Therefore, neutrophils could negatively regulate adaptive immunity through multiple pathways. Nevertheless, neutrophils are closely involved in keeping the balance between inflammatory and anti-inflammatory responses, which is very important to the host homeostasis and defending against pathogen infections. Unfortunately, we are not so clear how neutrophils efficiently coordinate this balance in details. To further clarify the functional plasticity and subpopulations of neutrophils in physiological and pathological situations may significantly help us to address this issue.

## Conclusions

Neutrophils, as the most abundant leukocytes, are heterogeneous cell population with high functional plasticity. In general, neutrophils act as the first defence line of immune system, which have a high potency and efficacy to sense and eradicate pathogen infections. Although historically they are considered as one kind of phagocyte of the innate immune system, more and more evidence has supported that neutrophils can also play an important regulatory role in adaptive immune response. Recent studies have claimed that neutrophils can promote T-cell activation and migration as well as DC maturation by multiple pathways. Neutrophils could acquire the feature of APCs to present antigens and result in the activation of adaptive immunity. The inflammatory microenvironment is the stimuli which can make neutrophils acquire the APC-like ability. Also, T cells can shape neutrophils to differentiate into APCs. Neutrophils are capable of modulating adaptive immune responses through interactions with T, B cells and possibly APCs. The role of neutrophils in B-cell immunity cannot be ignored. The production of neutrophils stimulated by G-CSF, BAFF, is critical for B cell maturation and survival. Interestingly, neutrophils can have negative effect on immunity. They can negatively influence CD4^+^ T cells as well as the interaction between some immune cells to supress the immune response. However, the detailed molecular mechanisms for neutrophils-mediated regulation on adaptive immunity still remain unclear. Further study is essential and of significance to unveil the detailed mechanisms under the regulation of neutrophils on adaptive immunity. On the other hand, the heterogeneity of neutrophils is recognized and more and more subsets of neutrophils were recently identified in humans and mice. However, physiological and pathological significances of these newly identified neutrophil subsets need to be further clarified in the near future. Recognizing the regulatory roles of neutrophils in adaptive immunity would promote us to re-consider the biological significance of neutrophils in physiological and pathological situations. Furthermore, the novel treatments of certain diseases, such as SLE and cancers, may be potentially provided based on the regulatory ability of neutrophils on adaptive immunity.

## Data Availability

Not applicable.

## Abbreviations

aGVHD: Acute graft-versus-host disease
APCs: Antigen-presenting cells
APRIL: A proliferation inducing ligand
BAFF: B cell-activating factor
CFA: Complete Freund adjuvant
dLNs: Draining lymph nodes
DTH: Delayed-type hypersensitivity
HEVs: High endothelial venules
HLA-DR: Human leukocyte antigen – DR isotype
i.p.: Intraperitoneal
ICAM-1: Intercellular adhesion molecule I
LNs: Lymph nodes
MHC II: Major histocompatibility complex II
MLR: Mixed leukocyte reaction
moDCs: Monocyte-derived DCs
MPO: Myeloperoxidase
MZ: Marginal zone
N1: Type 1 neutrophils
N2: Type 2 neutrophils
N_BH_: B cell–helper neutrophils
N_C_: Conventional neutrophils
NETs: Neutrophil extracellular traps
OVA: Ovalbumin
PBMCs: Peripheral blood mononuclear cells
pDCs: Plasmacytoid DCs
PDT: Photodynamic therapy
PMNs: Polymorphonuclear neutrophils
ROS: Reactive oxygen species
SLE: Systemic lupus erythematosus
TAMs: Tumor-associated macrophages
TANs: Tumor-associated neutrophils
TLR: Toll-like receptors
